# Ecology and conservation of socially learned foraging tactics in odontocetes

**DOI:** 10.1098/rstb.2024.0134

**Published:** 2025-05-01

**Authors:** Taylor A. Hersh, Daiane S. Marcondes, Gabriel F. Fonseca, João V. S. Valle-Pereira, Michaela A. Kratofil, Alexandre M. S. Machado, Shanan Atkins, Kyra R. Bankhead, Kiera McGarvey, Muhammad Mahmudur Rahman, Stephane P. G. de Moura, Fernanda Fecci, Mauricio Cantor

**Affiliations:** ^1^ Marine Mammal Institute, Department of Fisheries, Wildlife, & Conservation Sciences, Oregon State University, Newport, OR 97365, USA; ^2^ Center for Marine Studies, Universidade Federal do Paraná, Pontal do Paraná, Paraná 83255-976, Brazil; ^3^ Department of Ecology and Zoology, Universidade Federal de Santa Catarina, Florianópolis, Santa Catarina 88040-900, Brazil; ^4^ School of Animal, Plant and Environmental Sciences, University of the Witwatersrand, Johannesburg, Gauteng 2000, South Africa

**Keywords:** anthropogenic impact, animal culture, foraging specialization, odontocete, social learning

## Abstract

Culture—group-typical behaviour shared by community members that rely on socially learned and transmitted information—can drive animal adaptations to local environments and thus has the potential of generating specialized behavioural tactics to solve fundamental life challenges, including capturing prey. However, as human activities rapidly change the world in unprecedented ways, animal foraging cultures may no longer represent optimal solutions to local environments. Odontocetes (toothed whales, dolphins and porpoises) are of particular concern because they rely on learned, specialized foraging tactics in habitats highly affected by human activities. We present a global inventory of odontocete foraging tactics to evaluate their cultural underpinnings, vulnerability to human-induced threats and how this knowledge can inform safeguards. Our synthesis reveals a diverse repertoire—190 cases of 36 foraging tactics in 21 species—but highlights that linkages between culture and anthropogenic impacts are generally obscured by a dearth of data on individual identity, social associations and behavioural diffusion. By identifying global patterns, knowledge gaps and common threats to specialized foraging, our review can guide long-term research towards understanding their ecological and evolutionary drivers. This crucial first step towards designing policies that mitigate human impacts on marine habitats may ultimately protect the diverse odontocete behavioural repertoires that contribute to their survival.

This article is part of the theme issue ‘Animal culture: conservation in a changing world’.

## 1. Introduction

Behavioural specializations can significantly influence a population’s ecological and evolutionary dynamics, affecting resource use, population structure and trophic cascades [1,2]. These specializations arise from a combination of intraspecific competition, environmental and social heterogeneity and individual morphological and personality traits [3,4]. Increasing theoretical and empirical evidence highlights the role of social learning and culture in shaping specialized behaviours [4,5]. Through cultural transmission, behavioural innovations can spread rapidly within populations [6]. However, while behavioural specialization and cultural transmission amplify diversity, they also complicate conservation efforts—both the behaviours themselves and their ecological roles are worth safeguarding [7,8], but best practices for doing so are unclear.

**Figure 1.**
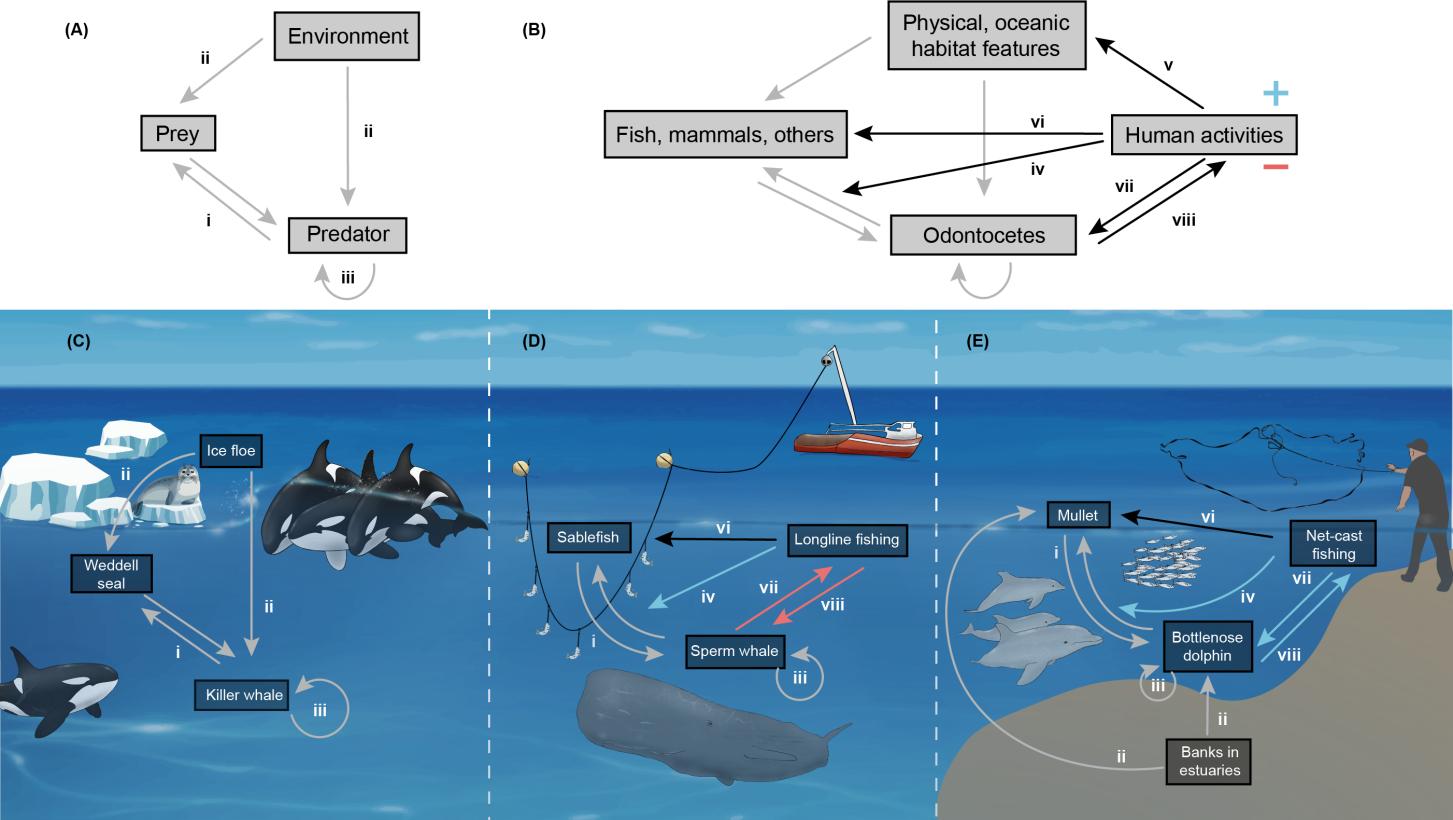
Ecological and behavioural drivers of foraging tactics in odontocetes. (A) A conceptual framework illustrating the interconnected, fundamental elements—environment, prey and predators—that make up a foraging tactic. (i) Predator–prey interactions are shaped by features of the physical environment, which influence the abundance, movement and behaviour of both (ii) predators and prey. Additionally, (iii) predators’ learning and other transmission processes (e.g. genetic, epigenetic) can further refine these interactions into specialized foraging tactics exhibited by a subset of the population. (B) The framework extends to odontocetes exposed to human activities; such activities can either hinder or promote specialized foraging tactics. Anthropogenic impacts may occur (iv) directly or indirectly—through alterations in (v) the physical environment, (vi) prey abundance or (vii) odontocete behaviour, which can generate (viii) feedback on the effects of human activities, further promoting (blue +) or hindering (red –) the development of specialized foraging. The three exemplar cases show the (C) absent, (D) negative and (E) positive influence of human activities on odontocete foraging tactics. (C) Cooperative wave-washing specialized foraging tactic exhibited by killer whales (*Orcinus orca*, ecotype B) in the Antarctic Peninsula where groups of killer whales swim in a highly coordinated way to create waves that dislodge Weddell seals (*Leptonychotes weddellii*) from ice floes [15], in the absence of humans. (D) Fish removal from demersal longlines by sperm whales (*Physeter macrocephalus*) spread through the Gulf of Alaska after the expansion of longline fisheries increased opportunities for sperm whales to prey on captured sablefish (*Anoplopoma fimbria*), causing catch and gear loss to humans and increased injury risk to whales [16]. (E) Cooperative foraging between bottlenose dolphins (*Tursiops truncatus gephyreus*) and artisanal net-casting fishers targeting migratory mullet (*Mugil liza*) in southern Brazil, where dolphins herd schools towards fishers in shallow estuarine waters and cue when and where fishers should cast nets in a synchronous tactic that benefits both species [14]. Original art by G.F.F. and D.S.M. Please see colour version of figure online.

The integration of behavioural [9] and cultural factors [10] into conservation practices has been gaining traction. Recent calls to action emphasize the need to conserve cultural diversity not only for its intrinsic value but also for its significant effects on individual fitness, population structure and resilience [11,12]. Cultural behaviours can delineate distinct population segments [10,12] and enhance a population’s capacity to adapt to environmental changes [13], which can be crucial for both individual survival and the long-term persistence of populations [14]. This is particularly true for behaviours related to solving the fundamental life challenge of finding and capturing food. Specialized foraging tactics can promote adaptive solutions to local environments and potentially alter predator–prey dynamics (figure 1A) [17]. However, anthropogenic activities increasingly disrupt animal behaviours [18,19], including foraging behaviours, and erode cultural diversity that has persisted for generations [20]. Paradoxically, these same human activities can also drive the emergence of new foraging strategies [21], highlighting the complex relationship (figure 1B) between human influence and animal cultures [22].

Odontocetes (toothed whales, dolphins and porpoises) offer a compelling case study for exploring the relationship between behavioural diversity, anthropogenic impacts and conservation. Their social [23] and cognitive abilities [24] create a fertile ground for culturally driven behavioural specializations to flourish [5,25]. Their diverse foraging repertoires, including tactics with cultural foundations [25], demonstrate their adaptive responses to local environments worldwide, including those altered by human actions. For example, killer whales pass down unique dietary preferences and hunting techniques through generations (figure 1C) [15], leading to the evolution of distinct ecotypes [26]; sperm whales rapidly learn from each other how to exploit human longline fisheries (figure 1D) [16]; and coastal bottlenose dolphins develop creative tactics for capturing prey in both natural environments and areas influenced by human activities (figure 1E) [27]. The spread and persistence of cultural foraging behaviours can profoundly impact population dynamics of odontocetes, leading to within-population distinctions that carry significant ecological and evolutionary consequences [10,23]. However, our understanding of the natural history, drivers and threats to these specialized foraging tactics remains incomplete, particularly regarding the cultural processes involved and their vulnerability to human impacts. This knowledge gap complicates conservation, as overlooking cultural factors can compromise the effectiveness of management strategies [7,28], but translating this understanding into practical actions remains extremely challenging [10,29].

Here, we present a global synthesis of foraging tactics in odontocetes, examining the evidence for their underlying drivers, the threats they face and the potential implications of cultural factors for conservation strategies. First, we conceptualize how specialized foraging tactics emerge from interactions among predators, prey, the environment and human activities (figure 1). Second, we conduct a scoping literature review to inventory and describe foraging tactics of odontocetes on a global scale. Third, we use the unified classification schemes from the International Union for Conservation of Nature (IUCN) and the Conservation Measures Partnership (CMP) [30] to evaluate the impact of human activities on socially learned foraging tactics, as well as the prevalence of existing and proposed conservation actions that target those threats. Finally, we discuss how the discreteness and evolutionary significance of cultural foraging specializations [10] could be integrated into future conservation efforts, and the challenges that doing so poses for practical actions. By identifying common threats to these specialized behaviours in marine predators, our review aims to guide long-term research on their ecological and evolutionary drivers, ultimately informing strategies to safeguard behavioural diversity.

## 2. Material and methods

### (a) Key definitions

Foraging is a behavioural state related to the search for, acquisition of and consumption of food. A *foraging tactic* refers to the immediate, situation-dependent actions taken towards the ultimate goal of consuming food and includes the decisions about where to search, how much effort to expend, which prey items to target and how to pursue, handle, capture or process an item before consumption [31]. While some foraging tactics are general, i.e. used by all individuals in a population, others can be distinctive and used by a subset of the population for reasons not exclusively linked to sex, age, or morphology [1,4,5]. We refer to the latter as *specialized foraging tactics*.

We are specifically interested in specialized foraging tactics that are, at least partially, driven by social learning and culture. *Social learning* is the acquisition of new information or behaviours that is facilitated by the observation of, or interaction with, another individual or its products [32]. Behaviours can be socially transmitted from parents to offspring (vertically), from non-parental individuals of the parental generation (obliquely) and/or from individuals of the same generation (horizontally) [33]. When a foraging tactic spreads through a population via social learning and is shared among a subset of individuals, it can be considered a cultural trait [25]. We adopt the broad definition of *culture* as ‘group-typical behaviour patterns shared by members of a community that rely on socially learned and transmitted information’ [34, p. 4].

### (b) Literature review

We conducted a scoping review of primary and secondary literature on odontocete foraging tactics (electronic supplementary material, table S1). We initially identified eight key secondary sources providing compilations of cetacean foraging behaviours [25,27,31,35–39] and extracted all cases of odontocete foraging tactics along with their original references. Through hand searches of Google Scholar, we expanded this list to include more recent peer-reviewed studies. When primary sources referenced additional foraging tactics, we added those tactics to our list. We excluded studies on foraging as a general behavioural state or on odontocetes in human care. In total, we analysed 281 peer-reviewed articles, book chapters and grey literature. This scoping review generated a comprehensive, but not exhaustive, list of foraging tactics in odontocetes (complete dataset available at [40]).

For each foraging tactic, we extracted a suite of metrics that are described in detail in electronic supplementary material, table S2. These included: species, location, foraging category, prey type, habitat type, tactic driver, whether the tactic is human-induced, whether information is available to identify individuals, and whether the tactic is shared. We then applied two ‘filters’ to this dataset of foraging tactics: the first to identify putative specialized foraging tactics and the second to identify which putative specialized foraging tactics could be cultural traits. We refer to the latter as *putative cultural foraging tactics*. At both filtering steps, we used inclusive minimum criteria that aligned with the definitions in §2.1 to ensure that no relevant data were excluded.

The minimum criteria that a foraging tactic had to meet to be considered a putative specialized foraging tactic were that individual identity information was available and that the tactic was shared by two or more individuals. For tactics meeting these criteria, we extracted demographic information of the individuals displaying the tactic (sex and age class) and whether there was evidence that the tactic was socially learned. If forager demographics had changed over time (e.g. owing to management actions [41]) then we used the most updated information.

We only considered specialized foraging tactics with positive evidence of social learning as putative cultural foraging tactics. For these tactics, we first recorded the type of evidence for social learning and the transmission direction, if known. Then, we investigated whether any of the corresponding references acknowledged anthropogenic threats to the tactic (i.e. threats to the predator, prey, environment or behaviour itself), as well as whether any references discussed existing or proposed conservation actions pertaining to the tactic. We classified both threats and actions according to the IUCN–CMP Unified Classifications of Threats and Conservation Actions [30]. A lack of acknowledgement of threats or conservation actions should not be construed as a true dearth of either. Our goal was to understand how often these topics were discussed in papers about foraging behaviour; we did not explicitly target papers about threats or management, but opportunistically recorded when these topics were mentioned. Similarly, if we knew of an existing threat or conservation action but it was not mentioned in any of the reviewed references, we did not include it in our dataset.

Finally, for putative cultural foraging tactics with quantitative evidence of social learning, we evaluated whether the available evidence suggests the behaviour can be used to assess the discreteness and/or evolutionary significance (electronic supplementary material, table S2) of potential conservation units [10]. Discrete units are demographically isolated, and evolutionarily significant units are repositories for unique information and behaviour that cannot be quickly reconstituted if units are lost. Evidence for differences in diet or foraging techniques that are stable (i.e. persist over multiple generations) and inherited (i.e. socially learned) will typically satisfy the criterion for both discreteness and evolutionary significance [10].

## 3. Results

**Figure 2.**
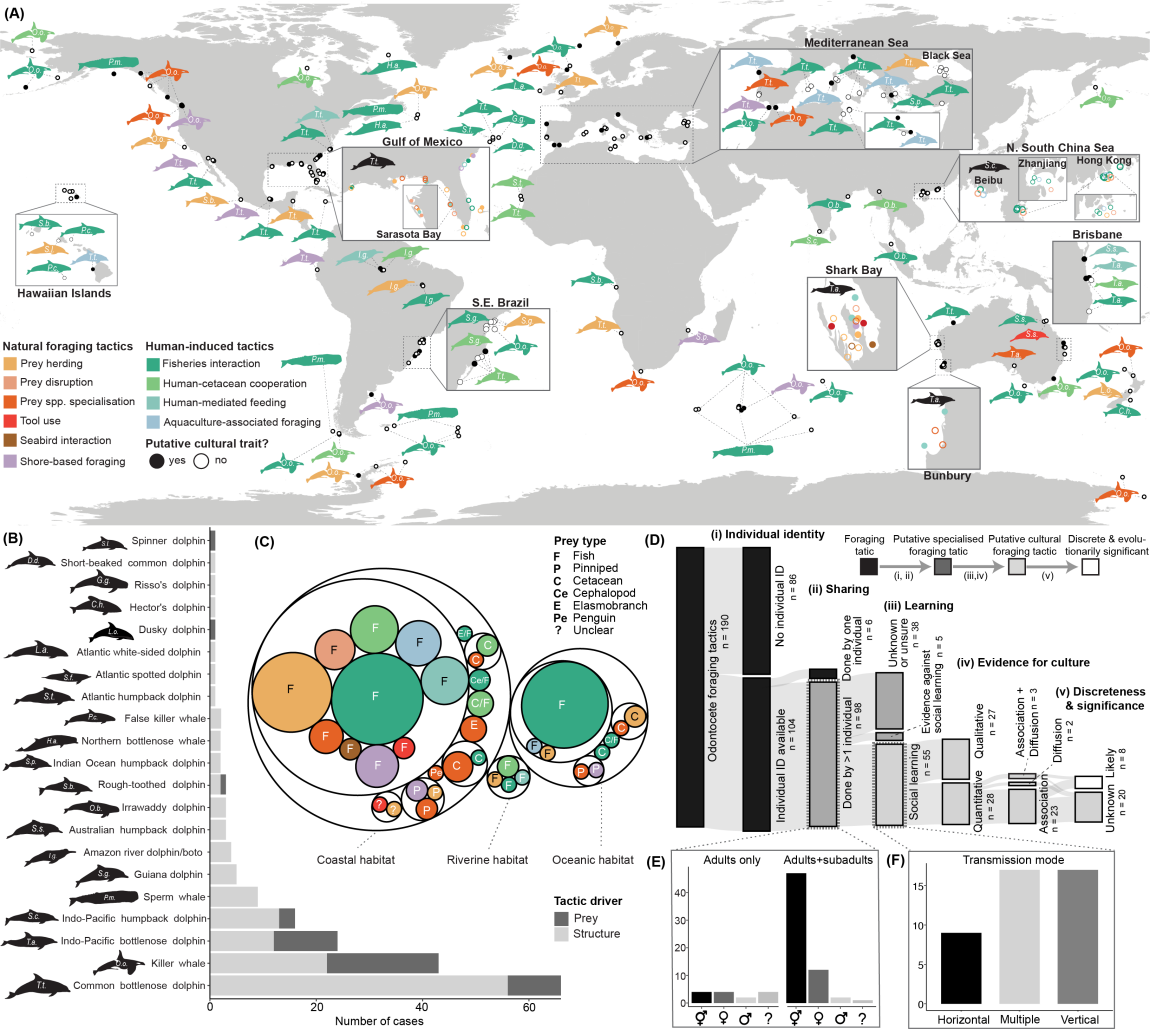
The global scope of odontocete foraging tactics. (A) Spatial distribution of 190 documented foraging tactics across 21 species, primarily in the Global North, categorized into 10 broad categories (warm colours: natural foraging tactics; cool colours: human-induced tactics). Putative cultural foraging tactics—those linked to individual identity and supported by evidence of sharing and social learning—are indicated by filled circles. Latitude and longitude were manually jittered for overlapping points and are approximate. Fundamental ecological drivers of foraging tactics for each odontocete species, including (B) whether the tactic primarily relies on the nature of their prey or physical structures (both human-made and natural), as well as the (C) main prey and habitat types (circles proportional to number of tactics, coloured by broad category). (D) The classification of foraging tactics (black) into putative specialized tactics (dark grey) and putative cultural tactics (light grey), based on (i) individual identity information, (ii) evidence of sharing among multiple individuals, (iii) evidence of social learning and (iv) evidence for culture in the form of association between social structure and tactic use and/or diffusion of the tactic through learning; cultural tactics are further evaluated on evidence for discreteness and evolutionary significance (white). (E) Proportion of specialized foraging tactics with known information on the individual identity, sex and/or age of foragers. (F) Proportion of cultural foraging tactics with known information on the social transmission mode. All variables mentioned are fully defined in electronic supplementary material, table S2. Cetacean silhouettes were generated from illustrations provided by Uko Gorter. Please see color version of figure online.

Our scoping review yielded 190 cases of 36 distinct foraging tactics (electronic supplementary material, table S1; complete dataset available at [40]) among 21 species of odontocetes across the world (figure 2A) . Of the 190 cases, common bottlenose dolphins (*Tursiops truncatus*, 34.7%), killer whales (22.6%), Indo-Pacific bottlenose dolphins (*Tursiops aduncus*, 12.6%) and Indo-Pacific humpback dolphins (*Sousa chinensis*; 8.4%) dominate (figure 2B). Among 10 broad foraging categories (electronic supplementary material, table S1), fisheries interactions (40.5%) and prey herding (17.9%) are the most common, followed by prey species specialization (9.5%) and human–cetacean cooperation (8.9%) (figure 2A,B). The less common categories are human-mediated feeding (5.8%), shore-based hunting (5.8%), aquaculture-associated foraging (5.3%), prey disruption (3.7%), tool use (1.6%) and seabird interaction (1.1%). Foraging tactics were mainly reported in coastal habitats (75.3%), followed by oceanic (22.1%) and riverine habitats (2.6%) and primarily target fish (85.3%) and marine mammals (8.4%) (figure 2C). Structures (both natural and anthropogenic) were a key driver of 74.2% of foraging tactics, and diverse human activities seem to, directly or indirectly, facilitate 62.1% of odontocete foraging tactics (figure 2B). Individual identity information, most often from photo-identification, was available for 54.7% of cases. Just 3.2% of all 190 foraging tactics had only been described for single animals; the rest had at least some evidence of being shared (figure 2D).

Of all cases, 98 met the minimum criteria—having both individual identity information and evidence of being shared among conspecifics—for being a putative specialized foraging tactic (figure 2D). While the sex of animals engaging in these 98 specialized foraging tactics was sometimes unknown (26.5%), we most often found evidence that both sexes participated (52.0%), followed by evidence of females only (16.3%) or males only (5.1%). Similarly, both adults and subadults participated in most specialized foraging tactics (63.2%), with a smaller proportion of tactics restricted to adults (14.3%) and none restricted to subadults (age class was unknown for the remainder) (figure 2E).

In total, 55 putative specialized foraging tactics had positive evidence of social learning and thus could be considered putative cultural foraging tactics (figure 2D). Reviewed papers specifically acknowledged culture as a potential tactic driver for 37 of the 55 cases. We found quantitative support for social learning in 50.9% and qualitative support in the remaining 49.1% of these 55 cases. Of the 28 cases with quantitative support, the evidence was most often (82.1%) based on analyses indicating a correlation between social structure and foraging tactic use. A much smaller number of these 28 cases provide quantitative evidence for the diffusion of the foraging tactic through the population (7.1%) or a combination of social structure and diffusion analyses (e.g. network-based diffusion analysis; 10.7%; figure 2D). In total, eight of the 28 cases had enough evidence to likely indicate discreteness and/or evolutionary significance of potential conservation units; examples include bottlenose dolphins foraging with marine sponges [42], bottlenose dolphins cooperatively foraging with artisanal fishers [14] and killer whales specializing on certain prey [43]. Of the 55 putative cultural foraging tactics, the social transmission direction was known or hypothesized in 43 cases (78.2%). Among those 43 tactics, evidence for vertical learning was most common (76.7%), followed by horizontal (51.2%) and oblique learning processes (14.0%)—note that some cases had evidence for multiple transmission directions, hence the sum exceeds 100% (figure 2F).

**Figure 3.**
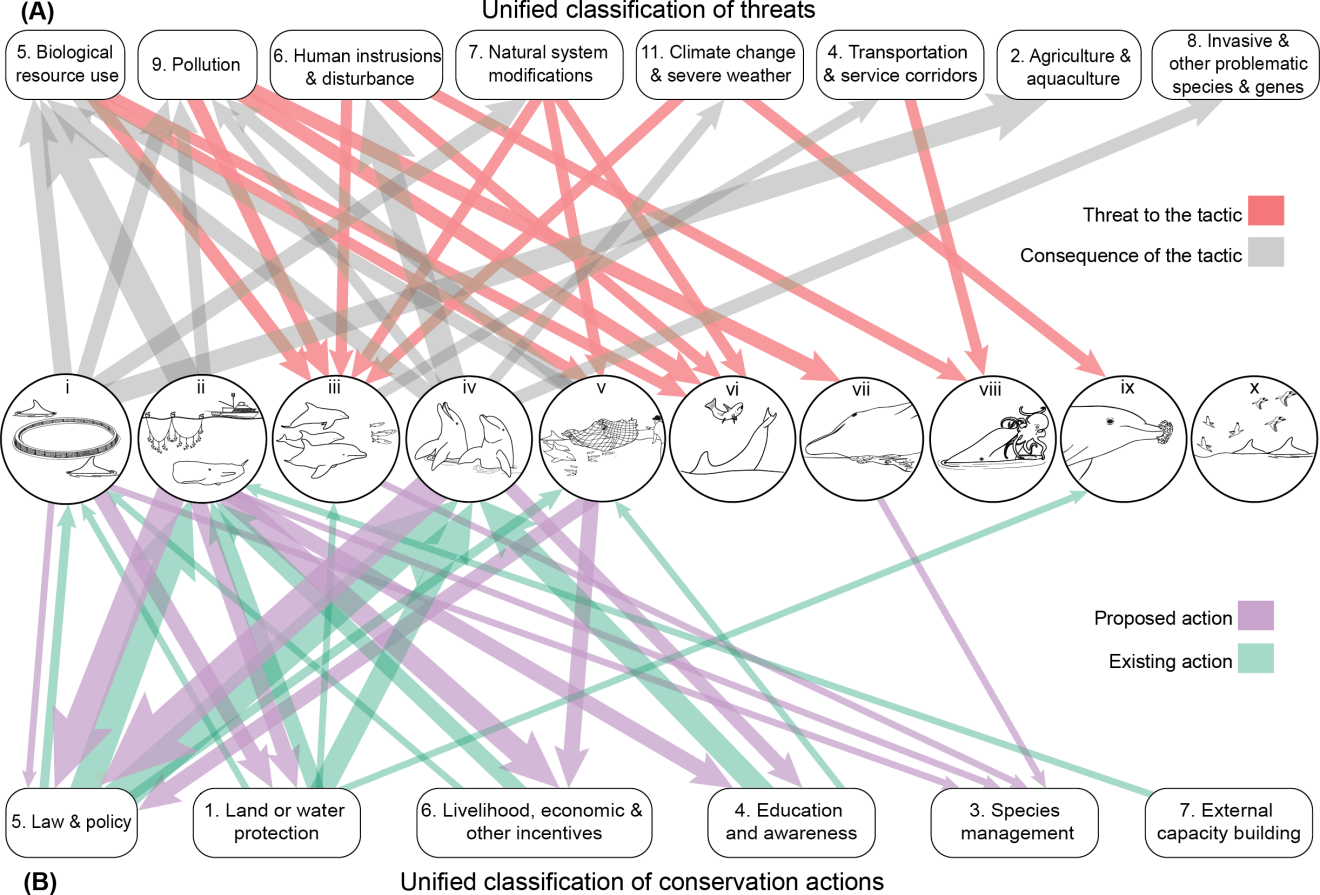
Threats to and safeguards of odontocetes’ putative cultural traits. Socially learned, specialized foraging tactics are (A) exposed to, and disproportionately affected by, a range of anthropogenic threats, and are (B) protected, or proposed to be protected, by a range of conservation actions. Threats and actions are numbered per the IUCN–CMP Unified Classifications. The foraging tactics are organized into 10 broad categories (electronic supplementary material, table S1): (i) aquaculture-associated foraging, (ii) fisheries interaction, (iii) prey herding, (iv) human-mediated feeding, (v) human–cetacean cooperation, (vi) prey disruption, (vii) shore-based hunting, (viii) prey species specialization, (ix) tool use and (x) seabird interaction. The tripartite network represents the threats to (red inward arrows) and threats that are a consequence of (grey outward arrows) the foraging categories and the existing (green inward arrows) and proposed (purple outward arrows) conservation actions reported in the reviewed studies. Arrows are weighted by the number of tactics within broad categories for which such threats or actions were reported. Original art by D.S.M. and G.F.F. Please see colour version of figure online.

Anthropogenic threats were acknowledged for 35 of the 55 putative cultural foraging tactics (63.3%) and single tactics often had multiple threats (figure 3). Of these 35 cases, human activities either affected the animals’ ability to perform the foraging tactics (25.7%; red arrows in figure 3A), or the execution of these tactics increased their susceptibility to anthropogenic impacts (74.8%; grey arrows in figure 3A). Under the IUCN–CMP Unified Classification of Threats, the most commonly recognized anthropogenic activities among these 35 cases fell under biological resource use (62.9%), human intrusions and disturbance (34.3%), pollution (25.7%) and natural system modifications (17.1%; figure 3A). Multiple threats were acknowledged for some tactics, hence the summed percentages exceed 100%. All threats under biological resource use fell into the same IUCN–CMP subcategory—fishing and harvesting aquatic resources—which includes bycatch, entanglement, fishing vessel strikes, overfishing prey and persecution by fishers. Similarly, for human intrusions and disturbance, all threats were associated with recreational activities (e.g. cetacean provisioning, recreational vessel traffic, unsustainable cetacean-watching tourism). Pollution threats spanned multiple subcategories and included household sewage and urban wastewater, industrial and military effluents, agricultural and forestry effluents, air-borne pollutants and excess energy (i.e. anthropogenic noise). Habitat degradation in the form of dams and water management use or other types of ecosystem modifications was sometimes reported. Threats were more often acknowledged for human-induced putative cultural foraging tactics (90.6% of 32 tactics) than for natural tactics (26.1% of 23 tactics). Prey herding was the foraging category impacted by the highest number of threat categories (*n* = 5), while human-mediated feeding led to odontocete susceptibility to the most threats (*n* = 5; figure 3A). In contrast, tool use and seabird interaction had few to no reported threats.

Similarly, conservation actions were acknowledged for 30 of the 55 putative cultural foraging tactics (54.5%; figure 3B). Among these 30 cases, authors typically discussed both existing and proposed actions (66.7%), but sometimes only mentioned one or the other (existing only: 20.0%; proposed only: 13.3%). By far the most common existing protection type (see electronic supplementary material, table S2) among the 30 cases was law and policy (70%; *n* = 21), with legislation (*n* = 16/21) and policies and regulations (*n* = 8/21) discussed much more frequently than compliance and enforcement (*n* = 3/21). Other existing protections among the 30 cases included: site/area protection (20.0%); awareness and communications (16.7%); substitution, in the context of livelihood, economic and other incentives (10%); and, for one case, alliance and partnership development (3.3%). We saw similar trends in proposed conservation actions (electronic supplementary material, table S2) among these 30 cases: authors frequently proposed legislation (26.7%) and policies and regulations (20.0%), but also highlighted a need for compliance and enforcement (30.0%). Authors also proposed conservation actions linked to livelihood, economic and other incentives, including substitution (20%); linked enterprise and livelihood alternatives (6.7%); and nonmonetary values (6.7%). Additional proposed conservation actions for those 30 cases included awareness and communications (13.3%), species management (13.3%) and site/area protection (10%). Once again, multiple actions were acknowledged for some tactics, hence summed percentages exceed 100%. Conservation actions were more often acknowledged for human-induced putative cultural foraging tactics (81.3% of 32 tactics) than for natural tactics (17.4% of 23 tactics). Fisheries interaction was the foraging category with the most existing (*n* = 4) and proposed conservation actions categories (*n* = 5), followed by aquaculture-associated foraging (3 existing, 3 proposed) and human-mediated feeding (3 existing, 2 proposed) (figure 3B).

## 4. Discussion

Our synthesis generates a global inventory of foraging tactics in 21 species of odontocetes, uncovering an array of behavioural diversity. In what follows, we discuss overarching trends in the distribution of natural and human-induced foraging tactics worldwide, including persistent biases in the most-studied species and regions (§4a). We also consider the unique conservation challenges that arise when foraging tactics are socially learned (§4b). Given the increasing pressure of human-induced threats to odontocete specialized foraging tactics, our synthesis emphasizes the need for dedicated, specific and timely threat mitigation efforts (§4c). Such efforts will be essential for ensuring the immediate survival of the species highlighted in our review. Long-term studies focused on gathering quantitative evidence for the cultural underpinnings of specialized foraging tactics are needed before conservation practitioners can implement culture-based targeted actions to safeguard this behavioural diversity (§4d).

### (a) Odontocete foraging tactics across the globe

Our scoping review reveals a wealth of behavioural diversity in odontocete foraging tactics. There is likely no single source for this within- and between-species diversity. Beyond ecological conditions and intrapopulation competition for resources [1,4], large brains and complex social structures are likely to further drive behavioural diversity in odontocetes [24,44]. Indeed, environment, large brains and behavioural innovation are linked in chimpanzees (*Pan troglodytes*), with behavioural diversity—including diversity in foraging tactics—highest in the most variable habitats [45]. Such environments are said to be characterized by ‘red noise’, where variation over long time-scales is large, and ocean environments are generally redder than terrestrial environments [46]. This environmental mutability sets the stage for extreme behavioural flexibility in odontocetes, including when it comes to developing solutions to the fundamental life challenge of finding food.

Odontocete foraging tactics could generally be characterized as either natural or human-induced. Natural tactics involve behavioural adaptations to exploit specific physical features of local habitats and/or the ecology and availability of preferred prey. For example, Guiana dolphins (*Sotalia guianensis*) use mangroves as barriers to trap fish [47]; common bottlenose dolphin groups intentionally propel themselves onto mud banks to capture fish they have driven out of the water [48]; and Indo-Pacific bottlenose dolphins use marine sponges to dislodge small bottom-dwelling fish from the sea floor [49]. In contrast, human-induced tactics often take advantage of anthropogenic structures that concentrate prey (e.g. trawl and longline fisheries, aquaculture facilities) and control prey movements (e.g. seawalls, dams) or modify human behaviour directly (e.g. provisioning, cooperative fishing). While all odontocete foraging tactics are susceptible to anthropogenic threats, the types of threats vary depending on whether the tactic is natural or human-induced (§4.3).

Certain odontocete species, habitats and regions are over-represented in our dataset, possibly reflecting their uniqueness and/or biases in the research effort. For example, bottlenose dolphins and killer whales account for 70% of cases, but are also the most intensively studied odontocete species [50]. Similarly, the emphasis on coastal foraging tactics over oceanic ones could be because coastal areas are more accessible to researchers, have more diverse physical features and/or are subject to more diverse processes than the remote and more stable oceanic waters [51], which may help to diversify coastal foraging tactics [27]. However, these biases could also stem from the historical long-term research efforts in and funding to the Global North. Unravelling the underlying drivers of biogeographical patterns in odontocete foraging tactics will require prioritized efforts in the Global South.

### (b) Specialized foraging tactics and putative cultural traits

Social learning is an advantageous strategy for the development of foraging behaviours in odontocetes [25,27,44]. Our synthesis reinforces this by gathering evidence for the link between social learning and foraging tactics for approximately 30% of all 190 foraging tactics reviewed. Vertical learning from mother to offspring was the main pathway reported for the transmission of specialized foraging tactics, highlighting female-based sociality as a cornerstone of cetacean societies and the substrate for cultural transmission [52].

Notably, 94.5% of the putative cultural foraging tactics were observed in bottlenose dolphins and killer whales, once again emphasizing the immense research attention that has been directed at these species. However, other species, including the Amazon river dolphin (*Inia geoffrensis*), the Australian humpback dolphin (*Sousa sahulensis*) and the sperm whale also exhibit human-induced socially learned foraging tactics. Even qualitative descriptions of social learning require long-term observations of behaviour; as such studies on odontocetes continue and data accumulate, we anticipate that social learning will be implicated in additional foraging tactics on this list.

At present, the dynamic interface of specialized foraging tactics, social learning and conservation is best illustrated by killer whales. Globally, killer whales are divided into ecologically distinct communities, called ecotypes (although the robustness of support for ecotype designation varies by region [26]). Sympatric ecotypes are specialized on different prey and exhibit specific suites of behaviours suited to that prey [26]. For example, eastern North Pacific killer whales are divided into transient, resident and offshore ecotypes, which specialize in salmonids, marine mammals and elasmobranchs, respectively [53]. These dietary preferences are socially learned within matrilines and can be quite rigid, even when individuals are faced with declining preferred prey populations and nutritionally suitable alternatives [54]. Orcas belonging to different ecotypes do not interbreed and may be undergoing incipient speciation as a result [53]; this makes ecotypes—not the species as a whole—the relevant unit for conservation, which has downstream implications for conservation and management actions (§4.4).

When dietary specialization is culturally driven, as is the case for orcas, models suggest that groups of animals can become more susceptible to extirpation [55]. However, not all cultural foraging tactics are as rigid as those seen in the highly structured, matrilineal societies of certain orca ecotypes. Bottlenose dolphins, which live in more fluid social systems, demonstrate that specialized foraging tactics can spread throughout entire populations without dividing the population into segments [56] and that existing segmentation can potentially be reversed through management actions [57]. Evaluating the resilience of odontocete groups exhibiting socially learned foraging tactics will require understanding the degree of reliance on the tactic, its transmission mode (vertical, oblique, horizontal) and its impact on shaping social structure [13,14]. This insight is crucial for developing effective conservation strategies but can only be achieved through long-term observations of individuals. Without such detailed knowledge, managers will face significant challenges in ensuring the persistence of culturally distinct populations, risking the loss of behavioural diversity [57] and potentially irreplaceable cultural knowledge [14,58].

### (c) Human-induced drivers and threats to putative cultural foraging tactics

Animals can adapt to human-induced environmental changes, often resulting in the loss or modification of existing behaviours and the emergence of new ones [18]. In non-human primates, for example, human activities can lead to changes in dietary habits and the development of new foraging tactics, simultaneously threatening existing cultural behaviours and promoting the development of novel ones [22]. As our synthesis shows, odontocetes have also adapted their foraging tactics to thrive in human-altered habitats, most notably in direct or indirect interactions with human fisheries. The nature of these interactions can vary from conflicts (such as removing prey from fishing gear) to neutral (scavenging and opportunistic foraging on discards) to mutually beneficial (cooperating with small-scale artisanal fishers) [35]. Given this preponderance of fisheries interactions, it is unsurprising that the most frequently highlighted threat to putative cultural foraging tactics included bycatch and entanglement in fishing gear, followed by vessel strikes and overfishing of prey. When odontocete interactions with fisheries are perceived as conflicts, persecution by fishers can also become a significant threat to animal health and life [59].

Most of these threats fall into the biological resource use IUCN–CMP category, which includes all deliberate and unintentional threats from the consumptive use of wild biological resources [30]. The prevalence of this threat category in our dataset highlights the ever-increasing potential for human–wildlife conflict [60]. Another IUCN–CMP threat category that we frequently encountered was human intrusions and disturbances, which includes threats from human activities that alter, destroy or disturb habitats and animals [30,61]. Human-mediated foraging tactics (i.e. provisioning and begging) belong to this category and present unique conservation challenges, as they create new ecological opportunities but may also cause animals to become overly reliant on human-provided food resources, sometimes at the expense of natural foraging behaviours [41,62]. Other potential examples of ‘anthropo-dependence’ [12] include neutral and mutually positive cetacean–fisheries interactions that shape the social structure of dolphins into distinct units. This is illustrated by the cooperative fishing between bottlenose dolphins and artisanal net-casting fishers that has persisted for generations [14], and the adaptation by some Indo-Pacific bottlenose dolphins that for decades specialized on feeding on trawlers’ discards but reverted to natural foraging tactics and socially reintegrated with the rest of the population after the fishery’s closure [57]. In such cases, effective management can become essential for animal welfare [41].

Reviewed papers were more likely to discuss threats for human-induced specialized foraging tactics compared with natural ones, likely because threats are extremely salient for foraging tactics centred on anthropogenic activities. Indeed, our results show that many odontocetes’ specialized foraging tactics are both induced and threatened by human activities, emphasizing the double-edged sword that is animal adaptation to human encroachment [35]. Additionally, certain IUCN–CMP threat categories, such as climate change and severe weather and natural system modifications, were conspicuously rare in our scoping review. This likely reflects challenges in forecasting climate and habitat change effects on ecosystems and animals, rather than a true lack of impact on cetacean foraging behaviour. For example, shallow-water foraging tactics that involve substrate manipulation, such as mud-ring and mud-plume foraging [63], may be compromised as sea levels continue to rise.

### (d) Safeguarding odontocete foraging behavioural diversity

Understanding behavioural variation and social learning is relevant for the successful conservation of odontocetes: while behavioural specialization may promote vulnerability, behavioural plasticity may promote resilience. Conservation efforts have traditionally targeted diversity at the species level because of its crucial role in ecosystem function. However, our growing understanding of how intraspecific variation and behavioural flexibility enhance populations’ resilience and influence communities and ecosystems [8,18] highlights the importance of considering finer population segments as conservation units [12,28]. The IUCN primarily assesses data for conservation efforts at global and regional levels, but tends to significantly underestimate, and thus inadequately safeguard, intraspecific variation [8]. Our synthesis highlights the diversity in inter- and intra-specific behavioural specialization in odontocetes, and below, we discuss two approaches to safeguarding this diversity: first, by interpreting trends in existing and proposed conservations actions, and second, by considering whether any of the foraging tactics in our review meet standard criteria for conservation unit designation [10].

Authors were more likely to acknowledge existing or proposed conservation actions for human-induced foraging tactics than for natural ones. The most common existing protections were legislation, policies and regulations, but enforcement to ensure compliance was rarely mentioned. As authors often pointed out [64], lack of enforcement undermines the efficacy of existing legal protections. For example, uncontrolled dolphin provisioning is common in Florida, despite such interactions being illegal under the U.S. Marine Mammal Protection Act [62,64]. Long-term education and outreach programs combined with pulsed enforcement efforts have been ineffective, which suggests that dedicated and sustained enforcement will be essential to protecting both dolphins and humans [62]. This example highlights an important point: ‘anthropo-dependent’ odontocete foraging tactics can be advantageous in the short-term (e.g. facilitated prey capture) but not necessarily in the long-term (e.g. reduced survival or reproduction [65]). Such tactics with the potential to be maladaptive [7] should therefore be discouraged rather than conserved, for instance, by working to alter human behaviour first [66]. Regardless, conservation practitioners and international policymakers should prioritize compliance with existing laws and have enforcement plans in place for any new legislation aimed at protecting foraging odontocetes.

Other frequently proposed (but rarely implemented) conservation actions fall under the IUCN–CMP umbrella of livelihood, economic and other incentives. These actions were often discussed in the context of fisheries interactions and human–cetacean cooperations—foraging tactics that engage directly with human dimensions. While interactions between cetaceans and human fisheries often lead to conflicts and economic losses [59], they can also foster cooperation. Cooperative foraging between humans and odontocetes showcases how these species influence each other’s behavioural adaptations for mutual benefit [36] while also highlighting how human and nonhuman cultures can be directly intertwined. The persistence of such cultural practices relies on protecting both the ecological factors (willing human and animal partners, shared prey and a conducive environment) and the compatible interspecies knowledge that guides synchronized foraging actions for mutual benefit [58]. Therefore, conservation strategies must be ethically grounded, explicitly involving Indigenous Peoples and local communities to thoughtfully incorporate their rich Local and Traditional Knowledge and safeguard their livelihoods [58] to ensure that human–cetacean interactions remain mutually beneficial.

Despite growing consensus on the importance of incorporating insights into animal culture into conservation efforts, concrete and practical methods for doing so remain nebulous. A recent proposed step forward is mapping out how cultural information can be used to assess the discreteness and evolutionary significance of potential conservation units [10]. Of the 28 putative cultural foraging tactics in our dataset with quantitative evidence of social learning (i.e. inheritance), only eight likely meet the discreteness and evolutionary significance criteria [10]. These eight cases were limited to long-term field research on bottlenose dolphins and killer whales. The remaining 20 cases very well might satisfy the aforementioned criteria, but additional years of monitoring are requisite to verify whether these foraging tactics persist for multiple generations. Other tactics that currently only have qualitative evidence of social learning, such as bottlenose dolphin mud-ring foraging [63], may be stable over time as well, but require quantitative analyses to confirm social learning.

## 5. Conclusions and future directions

Integrating nonhuman cultural heritage into conservation practices remains a complex endeavour. This approach needs to be rigorous and evidence-based to be efficiently implemented [11,29]. A persistent challenge is the paucity of detailed empirical data on animal culture across diverse taxa, which hinders the identification of common patterns and drivers that can aid the development of practical conservation tools. Here, we take a step forward by addressing that paucity for odontocetes—a taxonomic group for which culture can significantly drive individual behaviour, group interactions and population structure [25]. Through a scoping literature review, we characterized 190 different odontocete foraging tactics and evaluated threats and conservation actions for tactics with evidence of social learning. Our comprehensive yet not exhaustive review may have missed some odontocete foraging tactics but still offers a panoptic summary of existing knowledge in the field. Perhaps more importantly, this synthesis identified persistent knowledge gaps that must be filled before we can incorporate foraging cultures into conservation unit designation for most species.

During our scoping review, it became clear that information on forager demographics was often missing from papers about odontocete foraging tactics. For example, information on the identity of animals exhibiting specific foraging tactics was available for only about half of the 190 cases. Even when some identity information was accessible, the sex and age of animals were often unknown. Future research should prioritize collecting this baseline information because factors like sex and age can expose different demographics to unique threats based on the foraging tactics they use [67]. When faced with conservation triage, attending to threats that impact adult females could be prioritized, given this demographic’s essential role in calf rearing and, as our review emphasized, calf learning.

Relatedly, it was often unclear what proportion of a community or population exhibited a specific foraging tactic, which ultimately forced us to use a coarse, binary metric (exhibited by one vs. more than one animal) when evaluating whether there was evidence that a foraging tactic was shared. Understanding how frequently a tactic is employed by specific individuals relative to the rest of the population is requisite for evaluating tactic stability, which has implications for whether foraging tactic use can be incorporated into conservation and management decisions. Additionally, understanding a species’ social structure can provide managers with early hints of whether an emerging foraging tactic will crystallize or disappear. In matrilineal societies (e.g. orcas, sperm whales), both adaptive and maladaptive behaviours can spread, stabilize and become quite rigid, even as environmental conditions change [54]. In contrast, fluid, fission–fusion societies (e.g. bottlenose dolphins) appear to be able to ‘bounce back’ more quickly and adopt different foraging tactics in the face of change [57], but this also means that unique, generally positive foraging tactics, such as the cooperative human–dolphin fishery in Laguna, Brazil, may be quickly lost [14]. Accounting for social structure is thus crucial if managers want to discourage a maladaptive foraging tactic or encourage an adaptive one. For most foraging tactics, we thus must first resolve a lack of baseline data before we can productively consider how the *use* of a given foraging tactic can improve conservation outcomes for individuals and populations.

We found evidence of social learning for about 30% of foraging tactics, but expect the true proportion to be much higher based on ample evidence that social learning is an important process for cetaceans in general [25] and odontocetes specifically [23]. Detecting social learning is almost impossible without long-term, repeated observations of individuals; for that reason, we once again emphasize that funding for basic research and monitoring programs is essential if international policymakers and conservation practitioners want to improve conservation by accounting for odontocete culture. In the meantime, developing and implementing policies that address acute threats to odontocete foraging tactics are essential to protect animals and conserve behavioural diversity, both in the short-term and over the longer time periods required to study social learning [29]. Our review showed that certain categories of threats, such as biological resource use and human intrusions and disturbance, are more prevalent than others. However, the most significant threat to any given foraging tactic generally reflects features of the tactic itself (e.g. whether it is human-induced, whether it relies on a structure), meaning that threats can be very species- and location-specific. This suggests that local conservation strategies will be crucial for safeguarding behavioural diversity and should be prioritized.

Our synthesis supports the calls for incorporating nonhuman culture in conservation efforts [28,68] while cautioning that this concept should enhance, not necessarily replace, existing strategies [29]. Despite regular discourse in recent years on the merits of integrating culturally transmitted information into conservation unit designation [11,69], barriers and disincentives continue to challenge evidence-based conservation interventions, and practical recommendations for *how* to integrate nonhuman culture into said interventions have only recently emerged [10,70]. These new resources can, in theory, help us transition from acknowledging the existence of animal cultures to actively conserving them [70]. Culturally transmitted foraging behaviour is a prime candidate for assessing conservation units [10], but our review of the odontocete literature shows that most foraging tactics are undercharacterized. Even those tactics that are shared within a subset of the population through social learning—and so are likely cultural traits—typically lack the information needed to assess discreteness and evolutionary significance. Our best hope lies in directing resources and capacity towards addressing pressing local threats to the persistence of cetacean foraging tactics, thereby buying us the time needed to truly understand the drivers of such behavioural diversity and how much it impacts the survival of odontocetes. While it may take generations of odontocetes, and generations of researchers, to fully incorporate knowledge of cetacean foraging cultures into conservation, these efforts are worthwhile—not just to reveal the cultural significance of these behaviours but to ensure their persistence in the face of accelerating human impacts.

## Data Availability

The compiled dataset, supplementary material, and code to generate the core figures and analyses are available at [40]. Supplementary material is available online [71].
